# Blockchain and Demand Response: Zero-Knowledge Proofs for Energy Transactions Privacy

**DOI:** 10.3390/s20195678

**Published:** 2020-10-05

**Authors:** Claudia Daniela Pop, Marcel Antal, Tudor Cioara, Ionut Anghel, Ioan Salomie

**Affiliations:** Computer Science Department, Technical University of Cluj-Napoca, Memorandumului 28, 400114 Cluj-Napoca, Romania; claudia.pop@cs.utcluj.ro (C.D.P.); marcel.antal@cs.utcluj.ro (M.A.); tudor.cioara@cs.utcluj.ro (T.C.); ioan.salomie@cs.utcluj.ro (I.S.)

**Keywords:** blockchain, demand response, energy data privacy, zero-knowledge proof, energy flexibility

## Abstract

Nowadays, the adoption of demand response programs is still lagging due to the prosumers’ lack of awareness, fear of losing control and privacy of energy data, etc. Programs decentralization, by adopting promising technologies such as blockchain, may bring significant advantages in terms of transparency, openness, improved control, and increased active participation of prosumers. Nevertheless, even though in general the transparency of the public blockchain is a desirable feature in the energy domain, the prosumer energy data is sensitive and rather private, thus, a privacy-preserving solution is required. In this paper, we present a decentralized implementation of demand response programs on top of the public blockchain which deals with the privacy of the prosumer’s energy data using zero-knowledge proofs and validates on the blockchain the prosumer’s activity inside the program using smart contracts. Prosumer energy data is kept private, while on the blockchain it is stored a zero-knowledge proof that is generated by the prosumer itself allowing the implementation of functions to validate potential deviations from the request and settle prosumer’s activity. The solution evaluation results are promising in terms of ensuring the privacy of prosumer energy data stored in the public blockchain and detecting potential data inconsistencies.

## 1. Introduction

Decentralized management of energy systems is an emerging trend that facilitates the implementation of actions to self-supply the local energy demand and to intelligently manage the demand side by engaging prosumers in demand response (DR) programs to achieve high desirable smart energy grid goals such as resiliency, autonomy, and decarbonization. Generally, smart meters, dynamic energy pricing schemes, and home energy management systems should allow network operators, energy suppliers, and prosumers to define DR programs for promoting load flexibility shifting actions. Integrating demand-side management and DR into local energy systems will add the flexibility dimension to energy prosumers that can become active energy users in the envisioned future energy systems and models. At the same time, the DR automation will allow distributed system operators (DSOs) to acquire in a cost-effective manner the required flexibility and to integrate a larger share of renewable energy sources (RESs) to effectively stabilize the grid without compromising supply security and network reliability.

However, active prosumers participation is essential to assure a well-functioning DR program, while network operators and energy suppliers may have conflicting interests with respect to the flexibility management in an unbundled market. In Europe, despite the increased interest in this area, the implementation of DR is still lagging due to the prosumers’ lack of awareness and audit of the DR process, lack of comfort or privacy, and finally loss of control [1]. Vital requirements are still missing, and fundamental technical descriptions are not being accurately followed thus prosumers flexibility is currently ignored [2].

In this context, the adoption of blockchain technology for the implementation of DR programs may bring significant advantages in terms of transparency, openness, improving audit capabilities through peers’ consensus, and decentralization. The blockchain-based digitization of energy assets via tokenization could unlock the potential of energy flexibility while assuring at the same time the consideration of local operational constraints and prosumers’ personal preferences enacting the compensation and offsetting of energy-related behavior increasing their individual and community level of engagement.

**Figure 1:**
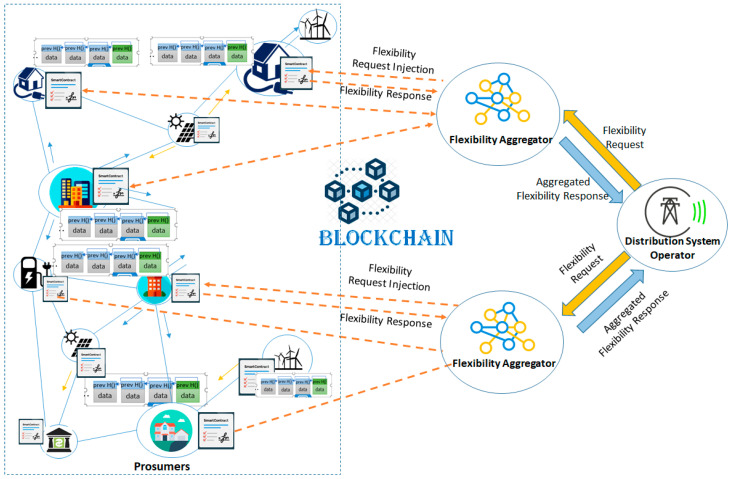
Flexibility based DR programs implementation using blockchain and smart contracts.

In such decentralized blockchain-based DR programs the prosumers can be involved through flexibility aggregators to offer and trade their flexibility in terms of loads modulation (see Figure 1). In a micro-grid, if a DSO identifies a congestion point and the associated grid connections, the aggregators are activated to leverage on the flexibility of the prosumers from their portfolio that are also connected to the congestion point. In this case, the DSO sends a flexibility request to which the aggregators respond by providing a flexibility offer. The DSO can accept one or multiple flexibility offers, can construct a flexibility order, and send it to aggregators that may adjust the load of their registered prosumers as required by the flexibility request.

Under a blockchain-based implementation, the energy consumption data of each individual prosumer is stored in the blockchain blocks as transactions while smart contracts define the rules for implementing the DR program. In such a decentralized DR management approach, each prosumer is enabled to offer its flexibility through smart contracts considering its enrolment to specific aggregators. The aggregators will inject individual flexibility request signals into smart contracts regulating individual prosumers energy consumption thus requesting them to adapt their energy profile by shifting flexible energy. Aggregators will evaluate the difference between the total amount of effectively activated flexible energy, normalized to each energy prosumer baseline energy consumption. If deviations are detected, penalties for prosumers are calculated by the smart contract while in the positive case the prosumer receives rewards considering the DR revenue rates and the level of energy demand profile adaptation to the DR event. The incentives of a prosumer are calculated by the aggregator using a flat rate established by the DSO than can be, for example, a daily revenue/discount rate for each kWh of energy shifted and that can be included on the common electricity bill.

The transparency of the blockchain-based system is most of the time a desired feature that brings openness and audit capabilities. However, in the energy domain, the prosumer monitored energy data are sensitive and rather private information thus, a private solution is required. In decentralized approaches, energy data are registered and stored locally in the blockchain using prosumers’ digital identities and then replicated and shared with all peers for validation. Considering that all energy transactions are duplicated and shared across the network peer nodes, it is important to apply solid methods for protecting this data. Moreover, when it comes to the implementation of DR programs presented above, the privacy concerns are listed as one of the main barriers which are hampering the full exploitation of the DR potential from smaller-scale electricity consumers. Indeed, nowadays there is a lack of secured interoperable solutions for assets flexibilization retrofitting and for supporting the shift from traditional centralized DR programs to more decentralized privacy-preserving ones driven by the consumer needs. In addition, with the proliferation of IoT (Internet of Things) energy meters deployment, consumers are mostly reluctant to give full control of energy data to aggregators / DSOs, since they would rather prefer to keep full control of their actions and data not to compromise their privacy being also supported by GDPR (General Data Protection Regulation).

The main contribution of this paper is the definition of a decentralized implementation of DR programs on top of the blockchain technology in which the privacy of the prosumer’s energy monitored data is assured by leveraging on zero-knowledge proofs (ZKPs) allowing at the same time the smart contracts to validate, on the chain, the prosumer’s activity inside the program. To ensure this aspect, our solution:

- replaces the monitored energy values of a prosumer, with a zero-knowledge proof that is generated by that prosumer;
- registers and validates the proof on-chain, being further used by the prosumer associated smart contract to evaluate and financially settle the prosumer’s activity;
- defines a validation function that estimates the deviation that is generated by a prosumer based on the monitored energy values from the requested flexibility.
- offers a ZKP implementation on top of a second-tier scalable distributed energy ledger proposed by us in [3].

The evaluation use case results show that our solution can ensure the privacy of the prosumer energy data that is stored in the blockchain and can detect inconsistencies in data that mean that someone tampered with the proof at the edge node level, before the energy transaction to be signed and deployed on-chain.

The rest of the paper is organized as follows: Section 2 describes the relevant related work in the area of blockchain, DR, and privacy preservation while Section 3 presents our ZKP solution for assuring the prosumers’ energy data privacy inside DR programs. Section 4 presents evaluation results, Section 5 analysis the proposed solution features in relation to problems of nowadays blockchain in implementations and finally, Section 6 concludes the paper.

## 2. Related Work

Smart contracts and distributed ledger technology (DLT) promise to offer a secure and reliable manner of exchanging information between energy prosumers, an infrastructure to automatize the flexibility provisioning and to implement decentralized DR programs [4]. They may support the construction of decentralized grid topologies that can pave the way for more localized energy systems enacting the democratization and transparent management of citizen energy communities. The envisioned benefits are the better load and flexibility management, secure energy transactions, near real-time tracking of generation and demand, efficient data aggregation, and optimized distributed energy generation management [5]. The goal is to construct more robust energy networks where every prosumer or flexible energy asset will be working independently or dynamically aggregated in virtual communities for improving the grid balance and stability. Several projects and literature papers address the decentralized implementation of DR programs and peer-to-peer flexibility orchestration using the blockchain technology. An important aspect is to provide energy transaction security in decentralized smart grids through blockchain enacted bi-directional energy trading [6]. The proposed platform allows consumers and producers to trade energy in a peer-to-peer network while the demand and the supply between two entities are matched through a mediator. Similarly, in [7] the authors propose a decentralized price-based DR solution in which prosumers can manage their consumption especially for adjusting their demand considering the local market price. Price is seen as a motivating factor for involving consumers in DR services and finally for avoiding the problems generated by the voltage stability issue. The blockchain-based energy transaction platforms can work in a decentralized fashion being also able to split the trading process into a call auction stage and a continued auction stage [8]. In this approach smart contracts attached to a transaction are developed on top of an Ethereum private blockchain and simulations show that the solution can achieve transaction settlement without the involvement of centralized entities. Decentralized blockchain mechanisms for DR programs management has been proposed in [9]. A distributed ledger stores the energy information acquired through smart metering devices and smart contracts are defined to capture the prosumers energy profiles, associated rewards/penalties, and the rules for optimizing the energy demand/production for a smart grid. In [10] the authors use blockchain and a welfare maximization mathematical model to create a dynamic pricing scheme for microgrids—DSO transactions which assure higher reliability in the ecosystem. The developments in the electric vehicle (EV) domain have generated serious grid stability and management problems. To deal with EV integration, approaches propose peer-to-peer electricity blockchain trading platforms to avoid power fluctuation without requiring a third-party broker entity [11]. Proof-of-Benefit (PoB) consensus primitive and smart contracts developed on the Ethereum platform also assure protection against attacks. Use cases have been proposed for validating a blockchain-based energy trading platform for EVs in small parking lots such as university campus [12]. The solution is developed on top of Hyperledger Fabric and enables selling/buying energy among participants, EV as buyers and charging stations as sellers, and in the same time manages to balance the university load demand. Blockchain-based green certificates systems such as I-Green [13], have been proposed in the context of consumer DR participation to promote the adoption of distributed renewable energy. This approach defines a new ratio incentive scheme and a proof of generation consensus protocol and is based on theories such as social norm and peer effect. Networked microgrids can take advantage of blockchain technologies and customized schemes to manage energy and financial transactions with the local distribution grid [14]. Auction-based trading mechanisms for peer-to-peer (P2P) energy trading and clearing mechanisms such as unified weight clearing can be used to construct a decentralized and autonomous energy trading process and creating coalitions of microgrids [15]. The scalability of integrating the real-time energy data coming from heterogenous smart meters into blockchain distributed ledger is an open issue well recognized in the literature. These advanced metering infrastructures require blockchain infrastructures to implement trusted peer-to-peer communication for protecting the exchanged information through distributed consensus mechanisms, encryption algorithms, and smart contracts [16]. However, the major drawback limiting blockchain adoption for smart grids is the high costs for data storage, hardware requirements for processing this data, and the small transaction throughput. We have proposed a scalable second-tier solution that combines blockchain technology with distributed queuing systems to register energy transactions less frequently on the chain and benefit from a Not only SQL(NoSQL) database scalability features for registering off-chain energy data [3]. This approach uses self-enforcing smart contracts and features tamper-proof and provenance tracking properties for the data stored on-chain.

The energy transactions of each individual prosumer may disclose behavioral consumer patterns and thus registering and disseminating such information may violate her/his privacy [17]. Data privacy-related research is focused on finding new solutions for protecting prosumers sensitive in energy transactions. Encryption algorithms such as Ciphertext-Policy Attribute-Based Encryption can be used to build a privacy-preserving trading model [18]. This algorithm can be combined with a Privacy-Preserving Blockchain Trading Scheme which uses a credibility-based equity proof consensus mechanism to maximize the protection of private information and improve overall security. For microgrid transactions, approaches combine agent theory, blockchain, hierarchical bidding, and Nash equilibrium for defining different bidding strategies that also ultimately assure the security of the whole transaction process [19]. Trusted bidirectional communication between energy prosumers, DSO, (transmission system operator) TSO, etc. can be also developed using existing protocols such as OpenADR or IEC 61850 [20]. A DR framework that assures data security using a blockchain infrastructure, smart contracts, and decentralized applications (dApps) for securing Aggregators-to-Prosumers transactions in the DR context proposed in [21]. This solution also uses OpenADR for supporting interoperability in terms of information exchange. In [22], an architecture that uses blockchain as a trust infrastructure to protect users’ privacy in the intelligent transportation systems domain is discussed and evaluated on the Ethereum platform. For scalability purposes, the authors use a hierarchical blockchain structure and define an incentive mechanism to encourage users to provide data.

Anonymizing data is an important issue when involving consumers and prosumers because the transaction information from the blockchain is public. Zero-knowledge proofs cryptographic techniques can assure privacy for verifying private data without revealing it in its clear form. Approaches have been already proposed starting from the loan market domain where ZKP blockchain platforms have been developed on top of zkLedger MIT Media Lab [23] up to specific ZKP such as zero-knowledge range proof that has been used in conjunction with blockchain for blockchain-based bank payments [24]. Another system that hides part of the transaction by making it private information is the HAWK system [25] which uses smart contracts that limit access to the data. The contracts are compiled to a lower level implementation that uses zero-knowledge proof algorithms or special graphs called snarks. Zero-knowledge proofs have been used by digital currencies to create anonymous transactions without the need of third parties. The goal is to implement zero proof knowledge algorithms on smart contracts to create an anonymous coin mixer for Ether unlinking transactions from the origin of the payment but still reveals payment amounts and destinations [26]. ZKP has been also adapted for enabling privacy-preserving authentication in the context of real-time charging operations for connected EVs [27]. An important decentralized identity management framework that provides the properties is the Web of Trust model [28], which was first introduced by the Pretty-Good-Privacy system to establish the authenticity and certification of identities and the data pertaining to them. These processes are entirely decentralized, i.e., entities can designate others as trustworthy by digitally signing their identity records (e.g., digital certificates). Thus, entities accumulate digital signatures from others that have deemed the former trustworthy. A verifier considers an entity’s data as “trustworthy” if one or more valid digital signatures, originating from other trusted entities, are met. Consequently, such solutions will provide a completely decentralized and fault-tolerant, in terms of both trust and security, identity management framework.

## 3. Zero-Knowledge Proof and DR Programs

To ensure the energy data privacy for the prosumers that are participating in DR programs we have implemented a solution based on ZKPs and blockchain. We have considered DR programs in which a contract in the form of a zero-knowledge (ZK) program specifying the flexibility request is agreed upon by both an aggregator and the associated prosumer. Whenever new monitored energy data is registered, the imbalance is computed based on the ZK program agreed, and the prosumer signs a proof of imbalance together with the associated transaction. The mined transactions trigger the prosumer associated smart contract execution, which evaluates the validity of the registered proof corresponding to the DR program. In case of successful delivery (i.e., the prosumer is accurately following the injected flexibility request), the prosumer would be rewarded (i.e., tokens would be allocated to its wallet), while in case of significant deviations between the request and the actual response are detected the prosumer would be penalized proportionally with the created imbalance.

In a regular blockchain-based implementation after a prosumer energy transaction containing the monitored energy data values is published and mined, every network participant has access to that information. Even if the identity of the prosumer is pseudo-anonymized due to the lack of mapping information between the prosumer’s address in the chain and the prosumer’s identity in real life, it could potentially become a security breach, since, if a malicious entity would be able to establish this relation, it could also extract behavioral patterns from the public energy profiles registered on chain.

To address this, we have implemented a privacy-preserving solution that uses ZKPs to hide the energy monitored data and requested flexibility profiles while registering on chain only the deviations and conducting a ZK proof validation to check that the deviation is correctly computed.

ZKPs allow one party, named the verifier to check if the other party, named the prover, has secret information without the prover to divulge the information by using four different functions: a key generator function, an input program function, a prover function, and a verifier function. The relation depicted in (1) shows the interaction among ZK functions for generating the keys. The key generator function G has as input the F-program function used to validate the secret and generates two keys: the proving key (PK) used by the prover to run the F-program function and to issue the proof that holds the correct secret, and the verification key (VK) used by the verifier to validate that the public value is indeed corresponding to the proof validating the undisclosed secret:

(1) $$G\left(F_{Flexibility}\right)=>\left(PK_{Flexibility},VK_{Flexibility}\right)$$

In our approach as generator function, we have used the ZoKrates toolbox for zkSNARKs on Ethereum [29] that given custom functions can generate the ZK keys to be used by each individual prosumer (see Table 1).

As validation rules implemented by the F-Program function ($F_{Flexibility}$), we have compared the flexibility request in a DR program communicated to a specific prosumer with its monitored energy data over the time interval of the response. Thus, instead of having the validation function to provide a true/false output, we have implemented a modified version where the output of the function is the deviation generated by the monitored value from the requested flexibility. The prover function P is used to generate the proof that holds the secret. The prover must provide as input the proving key, $PK_{Flexibility}$, generated by the key generator function G, the public information $H_{hour}$ and the secret $S_{EnergyData}$ which will be checked according to the validation rules provided by F. If the program F is successful, the proof is generated:

(2) $$P\left(PK_{Flexibility},\;H_{hour},\;S_{EnergyData}\;\right)=>proof$$

The verification function V is used by any player to check the integrity of the public information. The function receives as input the verification key, $VK_{Flexibility}$, together with the public information $H_{hour}$ (the hour associated to the proof) that will be validated against the proof published by the prover ($Proof_{deviation})$:

(3) $$V\left(VK_{Flexibility},\;H_{hour},\;Proof_{deviation}\right)=>true/false\;(3)$$

The proving key $PK_{flexibility}$ (see Table 2) will be used by the prosumer to generate the proof, while the verifier key $VK_{flexibility}\;$ will be used by a smart contract to verify the proofs provided by the prosumer, at the same time providing information about the deviation between the program request and its actual response. As a result, the only public information registered on chain is the hour $H_{hour}$ associated to the proof containing the output of the F-Program, that is the deviation measured by the prosumer.

### 3.1. Flexibility Request Communication

**Figure 2:**
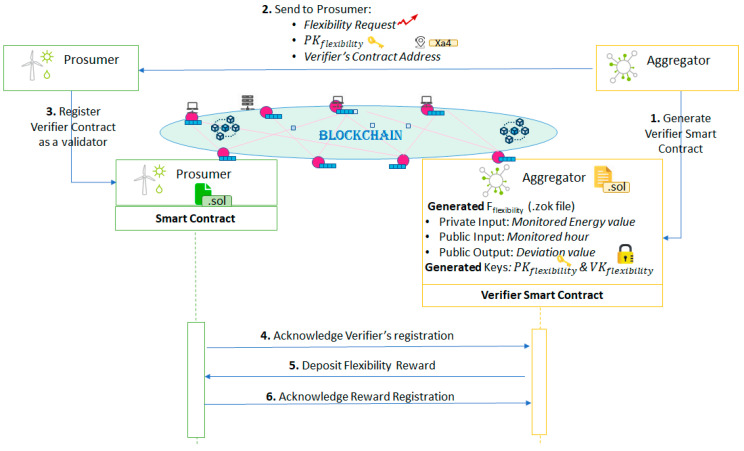
ZKP for enrolling prosumers in DR and assuring the privacy of data exchanged between aggregator and prosumers over blockchain.

In this section we present how the ZKP solution is used to assure the privacy of the data exchange between the aggregator and the prosumer for flexibility delivery. Figure 2 shows the success flow for enrolling a prosumer in the DR program augmented with ZKP specific elements.

**Figure 3:**
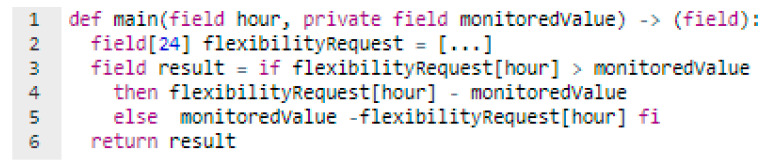
Function used by the aggregator for validating the prosumer response against the requested flexibility.

After the aggregator determines the flexibility request profile required from each individual prosumer, it generates the F-Program ($F_{flexibility}$) enforcing the DR program verification rules (see Figure 3). For each hour of the DR interval the $F_{flexibility}$ will compute the deviation as an absolute difference between the requested flexibility and the monitored energy value of a specific prosumer participating in the DR program.

Once the setup of the $F_{flexibility}$ program is complete, it will generate the prover key ($PK_{flexibility}$) and the verifier key ($VK_{flexibility}$). The prover key will be used by the prosumer to generate a proof, $Proof_{deviation}$ used to validate that according to the energy monitored value (now private information) registered by the smart meters, the deviation value publicly registered is indeed correct. As shown in Figure 3, for running the $F_{flexibility}$ program, the only specified public information are the hour and the output of the function (the deviation result) while the monitored value is kept private, thus leveraging on the ZKP mechanisms to validate the proof of the private monitored value against the public ones.

**Figure 4:**
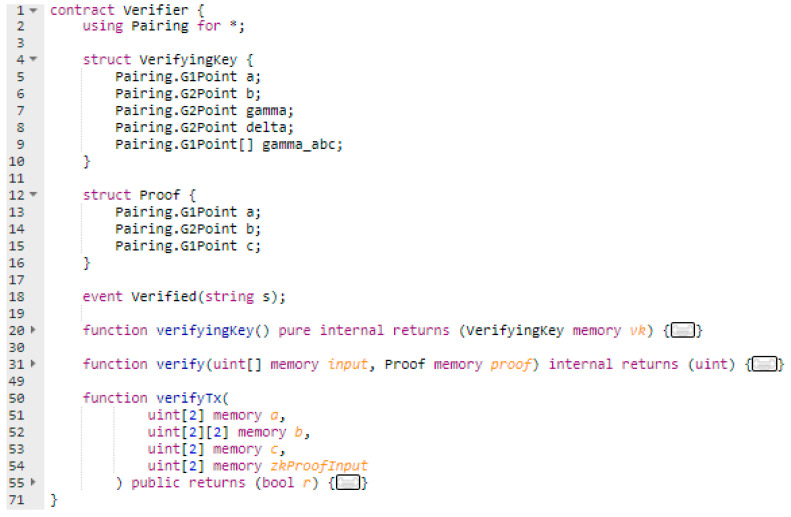
Aggregator smart contract using the verifier key for DR validation.

The verifier key is used by the aggregator associated smart contract as shown in Figure 4. The contract will be used to verify that indeed the energy deviation value made public by the prosumer and registered on chain is correct. This is done by using the $Proof_{deviation}$ generated by the corresponding $PK_{flexibility}$ based on the energy monitored value (i.e., this data is now kept private), and the time. More specifically the parameters used by the aggregator smart contract are: (1) three elliptic curve points (a,b,c) of the $Proof_{deviation}$ that make up the zkSNARKs proof and (2) $Proof_{deviation}$ inputs representing the public parameters specified by the $F_{flexibility}$ (i.e., zkProofInput [0] -> $F_{flexibility}$ input parameters: Hour, zkProofInput [1] -> $F_{flexibility}$ output parameters: Deviation Value).

The aggregator communicates to each individual prosumer: the flexibility request, the prover key,$\;PK_{flexibility}$, to be used by the prosumer, and the smart contract address used by the aggregator for verification. If the prosumer accepts the aggregator’s request, it will make this known by registering with the smart contract of the aggregator which acts as a validator in the prosumer associated smart contract giving permission to validate all the future energy transactions to be registered on chain.

Finally, the prosumer will send its acknowledgment to the aggregator by specifying the transaction receipt proving the aggregator’s smart contract registration as a validator. In return, the aggregator will deposit the token-based reward for the entire period in the prosumer’s contract and notify the prosumer of its action. From now, the prosumer is set up to register the proof of its activity on chain.

### 3.2. Energy Data and Response Deviation Registration

Once the setup and the prosumer’s registration with the flexibility request are completed, the prosumer energy consumption profile will be monitored and the deviations from the flexibility request profile are assessed during the DR program time interval.

The prosumer monitored energy data registration is based on the second-tier distributed energy ledger presented in detail in one of our previous works [3] on top of which we have integrated the ZKP solution. The ledger stores the prosumers deviations as energy transactions in a scalable and tamper-evident manner, which is a requirement for implementing decentralized DR programs. In our previous work, the energy data collected from an IoT metering devices associated with prosumer during a predefined time interval were hashed-linked back at prosumer level in the order in which they had been sampled and then were stored off chain in a distributed database. As a result, a single transaction was created and signed for each prosumer for such a time interval. The transaction was then published on blockchain containing the average energy value registered and the associated hash fingerprint generated. On blockchain, the average energy value registered was then used for further validation and implementing specific business logic for applications, while the digital fingerprinted transaction was linked hashed-back with the digital fingerprints of energy transactions generated for previous time intervals. The tamper-evident benefit assures that whenever historical data is requested, the digital fingerprint of the off-chain stored data will be computed and checked against the on-chain registered fingerprint. In case the hashes coincide, it is concluded that the off-chain real-time registered data has not been tampered with. Otherwise, further inquiries can be made to detect the exact interval where the data has been modified, thus leading to a tamper-evident system, where no changes can go unnoticed.

**Figure 5:**
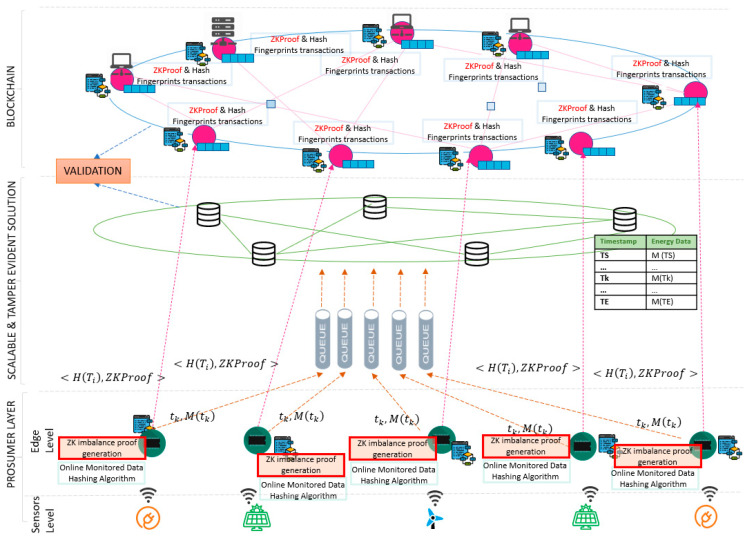
Second tier distributed energy ledger and zero-knowledge proofs integration.

Our ZKP approach is implemented on top of the scalable and tamper evident solution presented above. Figure 5 presents the layered architecture of the distributed energy ledger considered and how our ZKP solution is integrated to provide privacy over the monitored energy values:

- On the prosumer layer, the energy data is fetched from a sensor associated with a device identifier and a measurement type, the hashing algorithms are run and the connections to the off-chain and on-chain components are implemented;
- On the off-chain layer, scalable storage capabilities are provided and used for the real-time data received from the sensors. A queue-based asynchronous messaging system is used to ensure the data sequentially and system operation even in case of fluctuations in the monitoring data sampling rate;
- For the on-chain registration, the deviation between the monitored values and the flexibility request is computed and the ZK proof is issued. This information is signed and stored on blockchain together with the digital fingerprint computed based on the real-time monitored values in a hashed-linked back manner.

For each prosumer, we have considered that its associated energy metering device provides monitored energy data over a time interval P. The interval P is split in N smaller disjoint intervals $T_{i}$, where each interval $T_{i}$ is delimited by a start time and an end time $T_{i}=\left(T_{S}^{i},\;T_{E}^{i}\right]$:

(4) $$P=\cup_{i=1}^{N}T_{i}\;and\;\cap_{i=1}^{N}T_{i}=\emptyset$$

In each time interval $T_{i}$ there can be L ordered discrete timestamps in which sensors are sampling new data:

(5) $$T_{i}=\{t_{k}|t_{k}<t_{k+1},\;\forall k=0\ldots L-1\}$$

We denote the monitored value sent by a sensor at a timestamp $t_{k}$ as $M\left(t_{k}\right)$. From the sensor level, the monitored data value $M\left(t_{k}\right)$ is sent to the edge device at timestamp $t_{k}$. The edge device will forward this information directly to the asynchronous messaging system that stores the new sample data in the off-chain storage system in a sequential manner. At edge device level we had defined an online hashing algorithm that for each interval $T_{i}$ will compute the digital fingerprint of all the monitored data received from the sensor at each discrete timestamp from that interval. At the end of interval $\;T_{i}$, the edge device will sign and register the digital fingerprint of the monitored data on blockchain, thus ensuring an immutable log of this value. The blockchain will implement a decentralized storage algorithm that is responsible to store and compute a hash of period P, composed of all the digital fingerprints received for each interval $T_{i}$.

The zero-knowledge proof solution is integrated with the second-tier distributed energy ledger to provide privacy over the monitored energy values, while at the same time allowing the smart contracts to validate on chain the prosumer’s activity. As detailed in previous sections the ZK solution, replaces the aggregated energy monitored value, with a ZK proof which is generated at prosumer level. The proof is registered and validated on chain, being further used by the prosumer associated smart contract to evaluate and financially settle the prosumer’s activity. The mechanisms for computing the digital fingerprints remain the same, the fingerprints being registered in the prosumer smart contract, sealing them in an immutable log, and offering security features and the possibility of latter validation.

**Figure 6:**
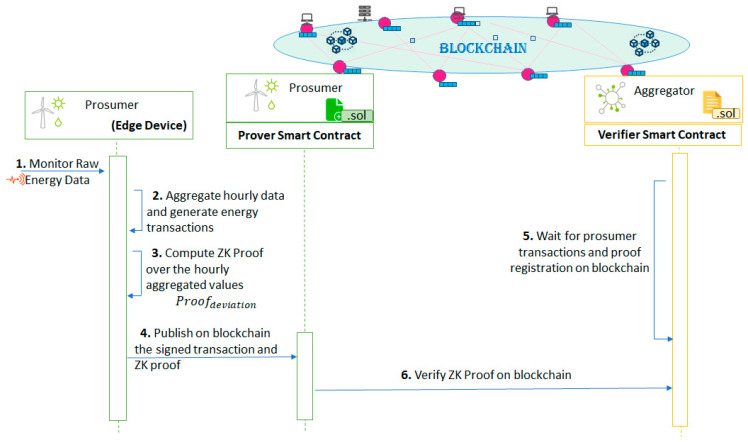
DR deviations detection process augmented with ZKP for data privacy.

Using ZKPs we will hide the registration of the compressed energy transaction value by computing the deviation off-chain and registering on the chain only the $Proof_{deviation}$ demonstrating that the computed energy deviation is indeed correct. The process of registering the energy deviation values from flexibility requests is shown in Figure 6. The monitored energy value received from the smart energy meters is firstly directly forwarded to the message queues, that ensure a scalable registration of the monitored values in the distributed database cluster.

**Figure 7:**
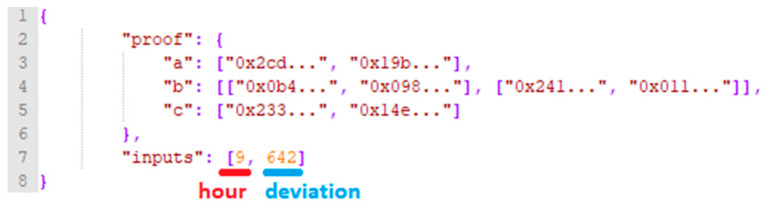
Snippet for energy deviation registration.

The monitored value is forwarded to the online hashing algorithm which periodically (at every hour) computes the average of monitored values together with the digital fingerprint of the values (obtained by hashed-linking back the real-time monitored values). The average monitoring value will be provided together with the corresponding monitoring hour and the proving key $\;PK_{flexibility}$ to the Prover Function, obtaining the $Proof_{deviation}$, and the deviation will be registered (see Figure 7). The edge node will sign a transaction intended for the corresponding prosumer associated contract, containing the digital fingerprint obtained and the $Proof_{deviation}$ together with the registered deviation value.

### 3.3. Smart Contract Interaction

The initials steps of the DR communication require the prosumer associated smart contract to be aware of the aggregator smart contract responsible to evaluate the near real-time prosumer activity inside the program. Furthermore, following the prosumer’s agreement to participate in the DR program, the aggregator needs to fund the prosumer smart contract with the token-based reward (i.e., incentives) for the delivery of the flexibility request. The prosumer smart contract will act as an escrow for the reward received from the aggregator. If the prosumer fails to register its progress or cheats during the delivery process, the aggregator will be able to retrieve the funds. Otherwise, if the prosumer succeeds to register his/her progress, the funds will decrease only if deviations from the initial agreement are registered, and at the end of the DR Program, the prosumer will have custody over the remaining funds.

**Figure 8:**
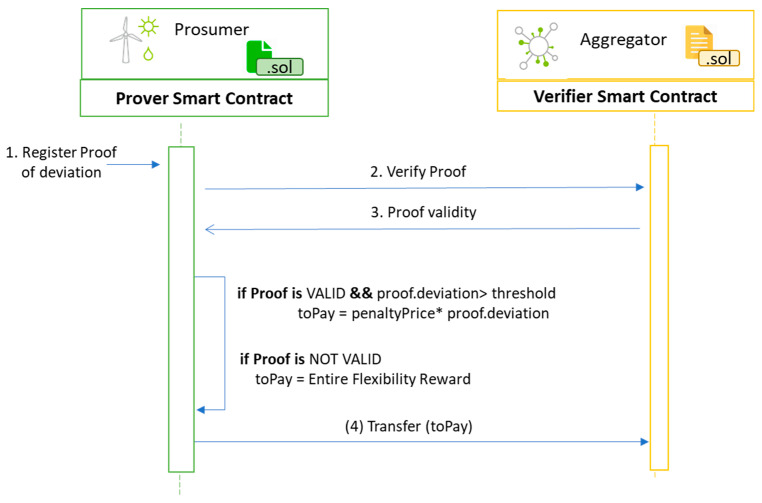
Privacy preserving smart contracts interaction for DR program implementation.

On the blockchain, the prosumer publishes the energy transaction containing the $Proof_{deviation}$ which is sealed in a new block, triggering a state update and the execution of the smart contract managing the prosumer energy consumption behavior. The interaction with the smart contract associated with the prosumer and aggregator for implementing the DR program is described in Figure 8.

Once a new transaction is published, the prosumer smart contract will enforce the validation rules upon the registered values. The prosumer contract will redirect to the verifier smart contract the proof registered by the transaction. If the proof turns to be valid and no deviation from the requested flexibility values is registered (i.e., the prosumer can comply with the request) the program associated token-based reward will remain into the prosumer wallet. Otherwise, the prosumer smart contract will compute the penalty proportionally to the deviation registered through the proof, and the penalty will be forwarded to the aggregator that initiated the DR program. If the proof is invalid (i.e., someone tampered with the proof, before signing the transaction which is possible only on the edge node, thus involving a malicious prosumer trying to cheat inside the DR program), then the prosumer will lose all funds received initially from the aggregator (i.e., they will be transferred back to the aggregator’s wallet). This will potentially discourage the malicious activity of prosumers inside the DR program while the aggregator may use the recovered funds to enter in an agreement with other prosumers from its portfolio for providing the needed flexibility in the remaining period.

## 4. Evaluation Results

**Figure 9:**
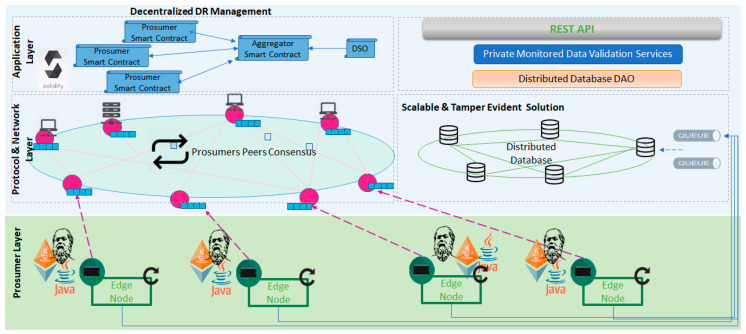
Prototype architecture and technologies mapping.

To evaluate the proposed approach, we have implemented a blockchain-based prototype for decentralized DR management that was enriched with the above described ZKP solution for preserving the privacy of prosumers data (see Figure 9).

The Prosumers Layer deals with the acquisition of energy consumption data which are delivered to a distributed data base using a queuing system for energy data aggregation for hourly energy transactions generation and to the associated Ethereum Node (local or remote) for periodically storing the private data values on-chain and issuing ZK proofs over the deviations from flexibility request and the registered energy values, using the ZoKrates tool.

The Protocol and Network Layer is built on a custom Ethereum [30] blockchain network where the smart contracts managing the business enforcement of the flexibility services are deployed. The contracts are implemented using Solidity [31]. For the blockchain setup, we have used the Proof of Authority as a consensus algorithm. To be able to compare our results with the public networks ones, while respecting the block times recommendations, we have considered the default mining time of 15 s and a gas limit comparable with that of the public networks of 8,000,000 gas per block.

The Application Layer features the self-enforcing smart contracts that are regulating the implementation of decentralized DR programs considered in this paper, and the interaction among the main involved parties, aggregators and prosumers. The validation services are required to annotate the monitored energy data that are stored in the distributed database marking the inconsistency found in respect to the already stored on-chain digital fingerprints. The Scalable and Tamper Evident solution module provides the implementation of our second-tier scaling scalable and tamper-evident solution for the aggregation, registration, and digital fingerprinting of the prosumers monitored data.

**Figure 10:**
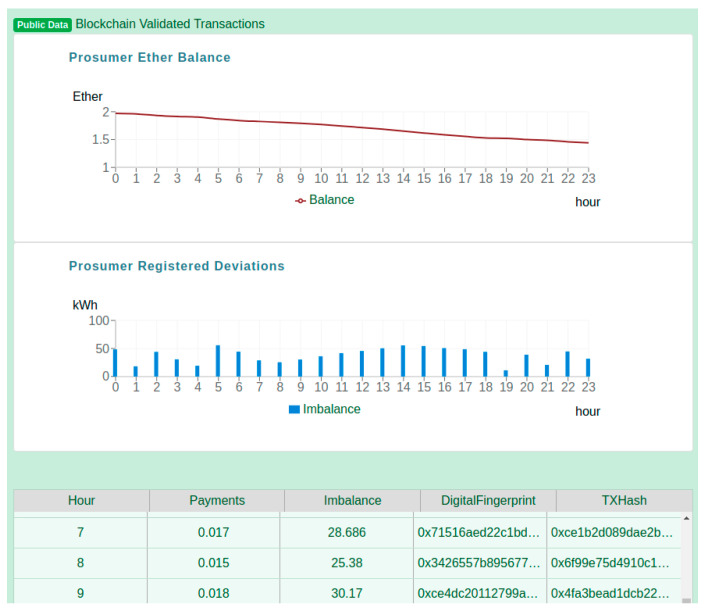
Prosumer’s public energy data registered in the blockchain platform.

For evaluation purposes, we have considered a DR program scenario in which a flexibility request is issued by an aggregator for a specific prosumer over a 24-h period (i.e., the length of the program). Figure 10 shows a view of the prosumer registered data, more specifically the public data that was registered in the blockchain and is made visible to all the network participants. There are two charts depicted. The first chart, “Prosumer Ether Balance” is showing how the balance of the logged-in Prosumer has changed during the day. The prosumer started the day having the contract pre-filled with ether, as a result of its agreement to participate in the DR program. However, during the day, the balance is slowly decreasing, due to the deviations that are registered between the monitored energy values and the flexibility request (both are private information). In the second chart, the “Prosumer Registered Deviations,” the bars show the deviations as the quantity of energy per hour registered in the prosumer associated smart contract. Finally, the table below the two charts, depicts the information registered hourly in the prosumer smart contract, and the energy transaction hash, offering the possibility to verify that indeed the transaction was successfully mined and validated by the network.

**Figure 11:**
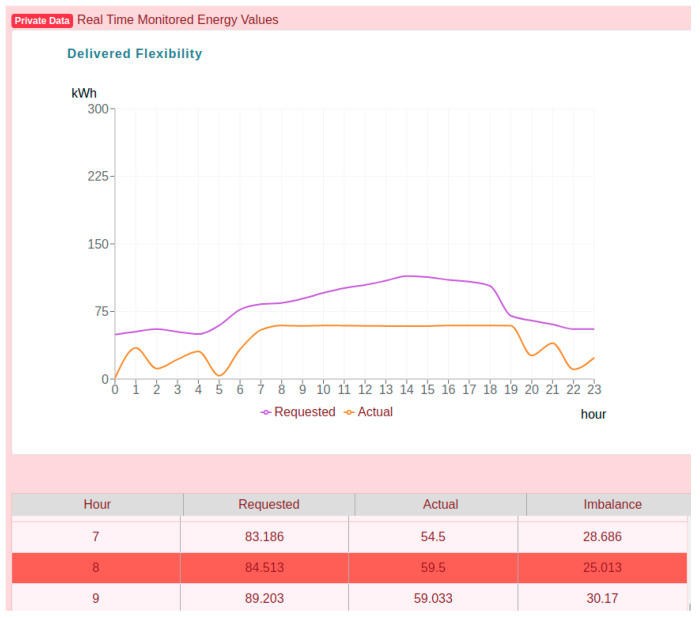
Private energy data available only for the prosumer.

Figure 11 shows the private energy data that is securely stored in platform, accessible only to the logged in owner prosumer. The chart shows the details of the flexibility requested profile received from the aggregator and the prosumer actual energy consumption profile monitored in real time.

In the table below the chart, it can be observed that indeed the deviations calculated by subtracting the flexibility request and prosumer monitored energy values are equal to the imbalance registered on chain. However, validation services identify the 8th hour aggregated energy transaction as being based on raw monitored energy values registered in the distributed database that may have been tampered since their registration.

**Figure 12:**
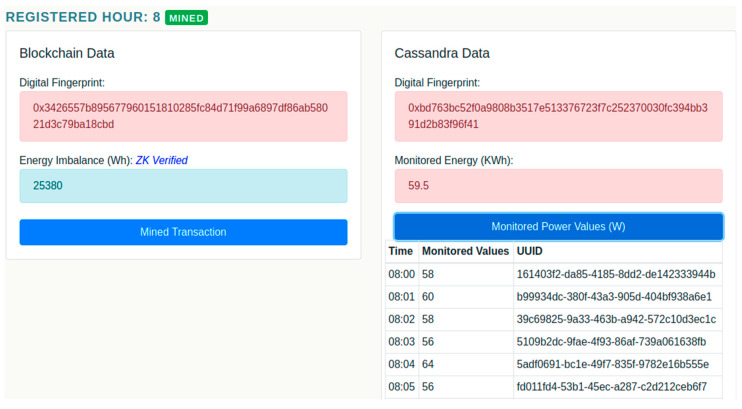
Validation of energy monitored values privately stored.

Figure 12 shows a detailed view of the validation carried out for the energy transaction registered and mined by the prosumer at hour 8 of the DR program. By running the digital fingerprinting algorithms proposed by us in [3] the tampered raw energy data is identified. However, this does not affect the deviation value registered on chain has successfully been validated using the ZKP, thus the client and the aggregator have been correctly evaluated during the delivery of the flexibility in the DR program.

**Figure 13:**
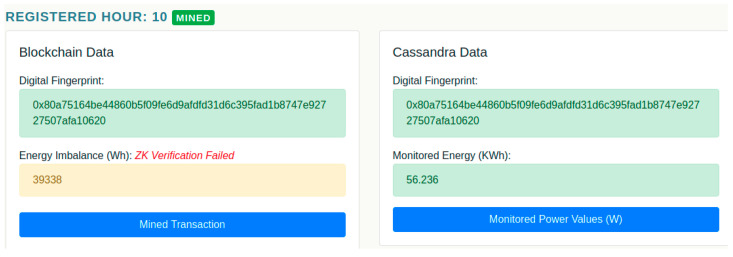
Inconsistency detected in the energy deviation registered on chain using the ZKP.

In the second scenario considered (see Figure 13), one can see that at hour 10, an inconsistency is detected regarding the deviation value publicly registered on chain. This means that, when the ZKP was registered in the prosumer smart contract, the aggregator smart contract have signaled an inconsistency between the hour, the declared deviation and the 3 elliptic curve points, meaning that someone tampered with the proof at edge node level, prior to the energy transaction being signed and deployed on chain.

**Figure 14:**
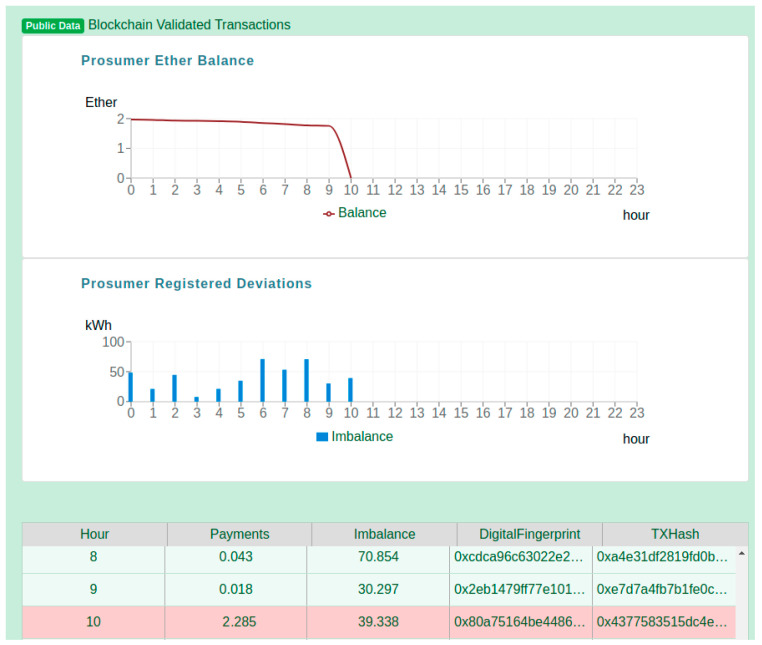
Public data on ZKP inconsistency detected and prosumer penalization.

A detailed view of the payments and activity on chain is shown in Figure 14 (i.e., the blockchain public information). Hour 10 is marked as being invalid according to the ZK Verifier key, and as a result in the “Prosumer Ether Balance” chart, it can be seen that the prosumer lost all its funds received from the aggregator with its enrolment in the DR program. The funds have been returned to the aggregator as a result of the prosumer misbehavior and attempt to cheat the network.

**Figure 15:**
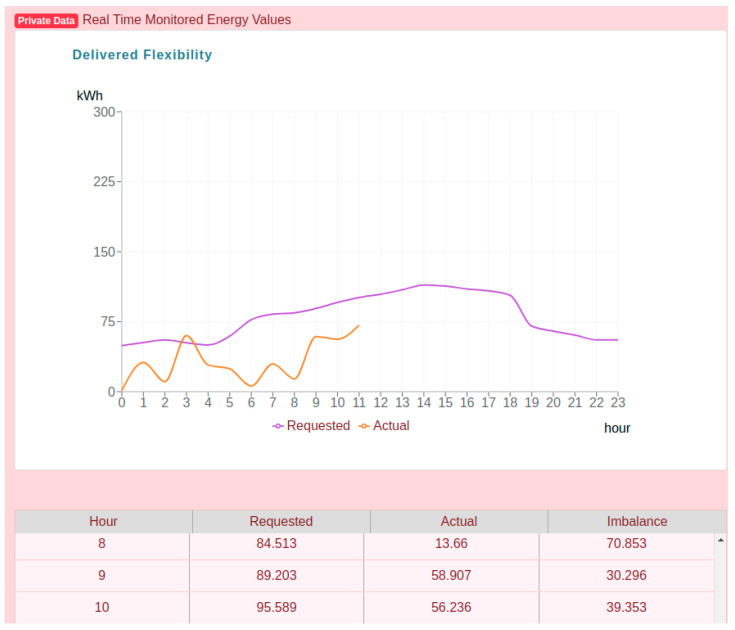
Prosumer private energy data information showing the stop of its participation in the DR program.

Figure 15 shows the monitored energy data of this prosumer, which is private. As can be seen the prosumer fails to comply and to deliver the requested flexibility values and moreover due to the detected inconsistency in the public data using ZKP it’s activity tracking is stopped, and it is no longer participating in the DR program.

## 5. Discussion

In this section, we analyze our solution and the obtained results in the light of some well-known problems concerning blockchain and its applicability to the energy domain and in particular the DR programs.

Most of the popular DLT solutions highlight the properties of transparency and openness to be major benefits brought by technology. Having open information about the transacting parties (pseudo-anonymous most of the time) and transacted assets, such systems may offer improved audit features [32]. Nevertheless, there are domains in which this is not required moreover in the energy domain one of the main obstacles for consumers’ engagement in DR programs is data privacy and anonymity. The recording of energy transactions of each prosumer may disclose behavioral consumer patterns and thus may violate her/his privacy [9]. Privacy preservation in a tamperproof manner is still an open research issue in the centralized approaches even though a lot of work was put in assuring privacy-preserving smart metering. Nowadays efforts are concentrating on providing a trustful bidirectional connection between the energy meters using protocols such as OpenADR. The IoT devices can provide private information registered in peoples’ houses, thus a highly secured and private system is required for storing the IoT data. In this context, our solution ensures the monitored energy data privacy in the blockchain, while not interfering with network-wide validation of the energy transactions. Instead of having the plain text energy values registered in the chain and validated by the smart contracts, in our solution, the validation is done off-chain using ZK mechanisms, and only the proof of validation is registered on the chain. As a result, even if malicious entities can join the blockchain network, no information regarding the actual energy consumption of specific prosumers is available, but only the proof that the validation was done off-chain is indeed correct for the specified imbalance. So even a malicious entity will join our system it will not be able to register the actual energy transactions thus no sensitive monitored energy data will leak. Although the imbalance value is registered on-chain and validated by the ZK-proof, an attacker could not extract any additional information since both the requested profile and the monitored profile are agreed directly between the aggregator and the prosumer beforehand.

Our solution based on ZKP and second-tier energy ledger may improve the DR programs audit and financial settlement by leveraging on peers’ agreement paving the way towards the implementation of micro-grid level improved consensus algorithms based on an adapted version of the two-phase commit and validation stakes. The Proof of Authority (PoA) consensus algorithm may still be used at blockchain tier (in an implementation based on Ethereum) for blocks validation while at lower network levels such consensus algorithm may be implemented empowering the peer nodes to agree on the validity of each energy transaction generated by each prosumer before is submission on the chain. This allows minimizing the risk of misbehavior due to malicious data provided by energy meters or altered by attackers on the communication network. Moreover, employing the ZK mechanisms will allow for the proof to be validated once it is registered on the chain. As a result, the actual validation will be subject to the benefits brought by the blockchain system (immutability, security, consensus, traceability) since the validation is done using a smart contract deployed on-chain. To render as valid the ZK-proof provided by a DR participant, the peer consensus must be achieved in the network regarding proof validity, thus each edge node will validate the prosumer signed transaction against the Verifier contract already registered on-chain for the associated DR signal.

Finally, since the edge application runs at the prosumer sight, there are some risks of losing the connection, thus the blockchain node could fail to connect to the rest of the peers in the network. Similarly, the real-time monitored data may fail to be forwarded to the distributed energy ledger. To mitigate these risks, a temporary storage solution is implemented at the level of the edge node which receives the monitored values from the sensors through an Message Queuing Telemetry Transport (MQTT) channel, applies the hashing algorithm as shown by us in [3], and stores it into a NoSQL database and periodically to forwards it to the blockchain node as a signed transaction containing the ZK-proof and the digital fingerprint. However, in case that the connection is lost, and the data fails to be sent through, the data is saved locally until the connection is re-established.

In terms of securing the smart meter to blockchain link, this is a very important open research direction not addressed by the current implementation. The most elegant solution is the development of new types of smart meters having this as a built-in feature. Research is underway for developing specialized hardware equipment for the edge nodes that can use safe cards [33] to provide key management and devices level security for blockchain integration. Anyway from the perspective of our provided solution to ensure the security at the edge level, each prosumer is required to have a node installed on-premises, either a full node on desktop computers or a light node on a small single-board computer (e.g., [34]) responsible to sign the transactions created based on the information gathered from associated IoT devices.

As in any digital system, there exists a risk of the private key/ passwords to be stolen. This is a well-known risk for all the participants of the blockchain network, regardless of the application or operations done on the blockchain.

Since each transaction registered on chain is signed and published by an edge node maintained by the prosumer, once a valid transaction is registered on chain, the prosumer smart contract will automatically verify whether the transaction is indeed signed with the prosumer private key (thus authenticating the origin/owner of the transaction). Once the authentication and authorization steps are successfully completed, the validity of the ZK proof is evaluated using the Verifier contract. However, if the prosumer intentionally modifies the ZK proof generated by the ZK prover program (for various reasons such as trying to cheat or to get a competitor prosumer penalized) and signs such a transaction with an inconsistent proof, that prosumer is automatically marked as malicious and excluded.

The scalability of integrating the real-time energy data collected by the smart meters into a blockchain distributed ledger is an open issue well recognized in the literature. Smart grid services have diverse requirements in terms of response time which impacts the granularity needed for energy data monitoring and the costs of energy blockchain integration. The second-tier energy storage solution had shown clear benefits in terms of throughput and response time considering Payment Channels and Private Setups as alternatives (more on this can be found in [3]). However, in terms of gas consumption, the introduction of ZK Proofs increases the amount of gas used per transaction, thus having an impact directly on the scalability of the solution. To evaluate this aspect, we have considered a private setup of the Ethereum network, having set the PoA as a consensus algorithm, the block gas limit set to 8,000,000, and default mining time of 15 s.

Upon introducing the ZK-proof validation per transaction, the gas consumption increases up to 680–769 gas for each transaction. Thus 11 transactions can be mined per block, considering a public blockchain setup dedicated to decentralized applications managing the consumption/production registration and evaluation. However, this bottleneck of a low number of transactions mined per block can be avoided by considering a larger periodicity for registering on the chain the ZK-Proof together with the digital fingerprints.

**Figure 16:**
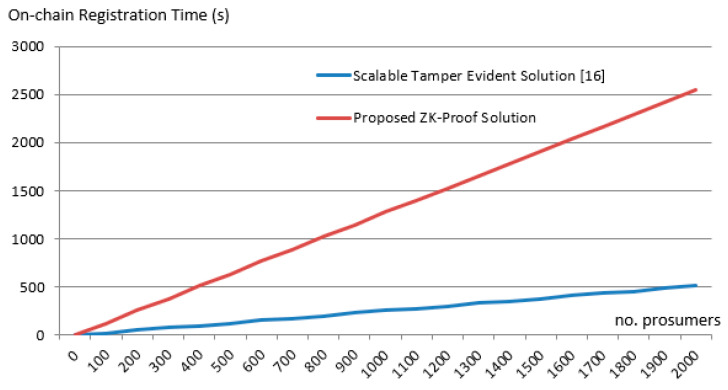
On chain registration time overhead brought by ZKP.

As can be seen in Figure 16 for 2000 prosumers, the minimum time interval for securely registering the energy transactions on-chain is approximately 9 min when the distributed energy ledger proposed by us in [3] is used without ZKP. With the ZK proof, the minimum time interval for registering the transactions and ZK-Proofs on the chain is 43 min. Even though the overhead is significant since with our solution the prosumer will submit 1 transaction per hour, we can still manage up to 2000 prosumers in a reliable manner.

## 6. Conclusions

In this paper, we have presented a solution for assuring the privacy of prosumer energy data in the context of decentralized DR programs implemented using blockchain and smart contracts. In the considered DR programs, an aggregator is responsible to inject the flexibility request in the smart contract associated with a prosumer and to validate the prosumer response and assess any potential deviation. The prosumers are managing their energy consumption behavior using smart contracts aiming to follow as close as possible the flexibility request. The proposed solutions integrate zero-knowledge proofs with the smart contracts defining the DR programs interaction rules to hide the prosumer energy monitored data and requested flexibility profiles while making public and registering on chain only the deviations and a ZK proof validation. The results are showing our solution potential in keeping the prosumer monitored energy data private while at the same time allowing the aggregators to conduct validations and identify potential deviations from the flexibility request and attempts on tampering the prosumer registered data.

## Figures and Tables

**Table 1:** Generator function for ZK proofs in DR programs.

Generator Function G	Generated Keys	F-Program	Validation Rules Implementation
zokrates	$PK_{flexibility}$ $VK_{flexibility}$	$F_{flexibility}\left(\begin{array}{c} H_{hour},\\ \;S_{EnergyData} \end{array}\right)$	field *result* =
			if *flexibilityRequest[H_hour_]* > *S_EnergyData_*
			then *flexibilityRequest[H_hour_]* – *S_EnergyData_*
			else *S_EnergyData_* - *flexibilityRequest[H_hour_]*
			fi

**Table 2:** Prover and verifier functions for ZK proofs.

Prover	Prosumer
Prover Function	$P\left(PK_{Flexibility},\;H_{hour},\;S_{EnergyData}\right)$
Prover Output	$Proof_{deviation}$
Verifier	Smart Contract
Verifier Function	*V*($VK_{Flexibility},\;H_{hour},\;Proof_{deviation}$)
